# A Narrative Exploration of Family Members' Perspectives of Life Story Phases Following Transition of an Older Family Relative Into Long‐Term Care

**DOI:** 10.1111/opn.70001

**Published:** 2024-12-05

**Authors:** Melissa Corbally, Orla Ffrench, Daragh Rodger, Rachele Ricci, Amanda Phelan

**Affiliations:** ^1^ Trinity College Dublin The University of Dublin Dublin Ireland; ^2^ Health Service Executive Dublin Ireland; ^3^ Dublin City University Dublin Ireland

**Keywords:** family relations, long‐term care, narrative research, nursing home, relatives, transition of care

## Abstract

**Background:**

The transition of an older person from a home environment into long‐term care is frequently unplanned and complex. Little is known about how relatives make sense of supporting the transition of their relatives to long‐term care.

**Objective:**

This study explored family members' narratives of the process of supporting the transition of their older relative into long‐term care.

**Method:**

Life stories of six relatives who supported transitioning their older family relatives into a nursing home were collected using open narrative questioning in accordance with the Biographical Narrative Interpretive Method. Data was analysed using a dialogic/performance analysis narrative analytic method.

**Results:**

Four key life story phases were identified: *before transition, crisis event, transition and after transition*. These phases varied in time duration and involvement of healthcare providers. The longest phase was ‘before transition’ where a process of slow deterioration became more apparent to the participants retrospectively. This was followed by the shortest period ‘crisis event’ where the older person was admitted to tertiary care. Two permeating themes: *family dynamics* and *knowledge/understanding* underpinned all life story phases.

**Conclusions:**

Relatives' knowledge, family dynamics and positioning of self‐informed the duration of the life story phases of participants as they navigated the transition. Understanding nuanced differences in relatives' life story phases highlights how timing of information provision can affect the emotional adjustment of relatives experiencing this challenging process.

**Implications for Practice:**

Both community and gerontological nurses' offer real potential to provide tailored and effective responses to relatives depending on each life phase. Sequencing of information appropriate to the life phase could potentially ease the stress associated with transitioning to nursing home care, possibly preventing a crisis event from occurring. Anticipatory conversations also offer potential to alleviate relatives' concerns through life story phases.

**Summary**

- What does this research add to existing knowledge in gerontology?
  ○ This narrative study identifies four distinct life story phases relating to relatives' experiences of the process of supporting the transition of their older relative to long‐term care.
  ○ Life story phases vary in time duration and the involvement of health care providers varies with each phase.
  ○ Health care providers were most influential during the ‘crisis event’ phase but had influence throughout all phases. However, there is scope to increase support for relatives in this transition.
  ○ Family structure and dynamics continue to influence the transitioning process.
- What are the implications of this new knowledge for nursing care for and with older adults?
  ○ This study identifies that relatives' knowledge needs vary throughout life story phases related to transition support.
  ○ Nurses in all settings can assist with enhancing information and knowledge before, during and after the transition phases as well as supporting the psychological and life world adjustment for relatives.
  ○ Nurses have the potential to provide targeted information and support during the ‘Before’ phase highlighting the ‘creeping up’ process of escalating care needs and possibly preventing ‘crisis’.
- How could the findings be used to influence practice, education, research, and policy?
  ○ Educators should incorporate the influence of societal expectations and previous promises made by relatives to their older relatives into educational resources relating to gerontological nursing
  ○ Findings suggest that greater decision‐making support as well as plain language information guides for relatives would be helpful in assisting them transition their older family relative into long‐term care.

## 1 Introduction

Globally, there is a dramatic increase in the number of older people (Kalache, Barreto, and Keller 2005; Lowenstein 2005; World Health Organisation (WHO) 2022). Whilst this is a triumph in relation to the longevity of humans, it places a responsibility on society to re‐orientate care to ensure the quality of life is enjoyed by older populations. As people age, there is a higher risk of functional and cognitive challenges and nurses are ideally placed to respond effectively to maximise quality of life in all settings. In the community setting, compensatory measures may also be provided by family, other caregivers, friends and healthcare professionals depending on a person's need. Examples of compensatory measures include assistance with dressing, mobilising or providing nutrition which are dependent on a person's self‐care status (Orem 2001). In many countries, gerontological and community nursing with a gerontological focus provide essential compensatory supports to enable ageing in place. In Ireland for example, Public Health Nurses and Community Registered General Nurses have a geographical caseload from cradle to grave, both of which include a substantial remit with the care of older people.

International evidence illustrates both community‐based and gerontological nurses not only have a role in patient care, but they also play a key role in supporting families, providing informal care to relatives and enabling them to remain in their communities for as long as possible (Leichsenring et al. 2013; Centre for Policy on Ageing 2014). Informal caregiving represents unpaid support and may include activities such as assistance with mobility, nutrition, hygiene, housekeeping, health services engagement or medication management (Kong et al. 2021). While many caregivers report positive aspects of caregiving (Quinn and Toms 2018; Yuan et al. 2023), there can be a negative impact on their health and social lives (Polacsek and Woolford 2022). However, when a person's needs surpass care provision capacity, a transition to long‐term care (LTC) may be considered (Kalache, Barreto, and Keller 2005; Kane and Kane 2005; Abdi et al. 2019; Sacco et al. 2022). This process has been identified as emotionally fraught for older people while relatives can experience conflicting emotions such as relief on one hand and guilty feelings on the other (Argyle, Downs, and Tasker 2010; Polacsek and Woolford 2022). This paper argues that relatives of older persons navigate through phases in their life stories of care and transition and that gerontological nurses as well as community nurses with a gerontological focus offer real potential to provide targeted and impactful responses to relatives depending on their life story phase. Whilst there is evidence in existence regarding older person's experiences of transitioning into LTC (e.g., O'Neill et al. 2020), less is understood about how family members make sense of these complex and challenging experiences. Consequently, this study explored relatives' narratives on life phases related to the process of supporting the transitioning of an older relative into LTC.

## 2 Method

### 2.1 Design

Nursing occurs within an oral narrative culture where stories shape care decisions, practices and experiences. Narrative Inquiry is a multifaceted, methodological perspective which seeks to understand more about the inherent complexities of human storytelling. It enables a more holistic exploration of participants' subjectivities and lifeworld as they evolve over time (Riessman 2008; Victor 2009) and is underpinned by two main assumptions. The first assumption is that narratives represent a human sense‐making process of lived experiences. The second assumption is that narratives are a means of social construction where meaning and reality are configured through the medium of language (Casey et al. 2016). Whilst these are not mutually exclusive, elements of both are often found in narrative research studies. Riessman's (2008) dialogic/performance analysis (the method utilised in this study) attends to both methodological assumptions appreciating temporality (time), content, context and linguistic positioning (how people talk) in its analysis. This approach also draws from social constructionism (Berger and Luckmann 1966) and the work of Goffman (1959) who recognise that individuals perform their identities in human interactions (such as nurses interacting with family members). Because the topic of study was experiences over time which were dependent on human interactions and communications, a dialogical/performance analysis approach which appreciates the duality and performativity in human communications was felt to be particularly useful (Riessman 2008). Dialogic/performance analysis facilitates the exploration of ‘when’, ‘why’ and ‘how’ stories are told in particular ways in addition to exploring ‘what’ was said (e.g., in traditional thematic analysis). Further descriptions outlining the phases of analysis are contained below.

### 2.2 Sampling

Recruitment letters were sent to all relatives who had supported the transition of an older relative into a large nursing home within a 3‐month period prior to January 2018. Six participants (male (1) and female (5)) participated in the study. All of the participants (one was a husband and wife who were interviewed separately) provided care to their older parents.

### 2.3 Data Collection

Open narrative questioning was used to collect data. This biographical narrative interpretive interview technique (Wengraf 2001, 2014) was developed as a means to empower participants to frame and shape their own narrative in their own time. Each interview began with a Single Question aimed at Inducing Narrative (SQUIN), adapted from Wengraf (2001, 2014). All participants were asked the same SQUIN which was: ‘As you know, I'm researching the experiences of family members who have been involved in the decision to move their older relatives into long term care. So please can you tell me your story, all the events and experiences that were important for you personally up until now. There is no rush, you can start wherever you like. I'll listen first, I won't interrupt. I might take some notes in case I and will take some notes if I have any further questions later’. The interview ended when the participants said they were finished. Following a short break, any clarifying questions related only to the SQUIN content were asked by the researcher. Interviews lasted between 22 and 79 min and were digitally recorded and transcribed.

### 2.4 Ethical Considerations

Institutional ethical approval for the study was granted by the Research Ethics Committee of Dublin City University in March 2017 (Reference: DCUREC/2016/207). Access to the site was granted by the Director of Nursing. The identities of participants were replaced by pseudonyms at the point of transcription.

### 2.5 Data Analysis

The analysis process was constituted of three phases (two main phases with an additional phase of collective interpretation underpinning both). The first phase focused on the analysis of individualised participant stories, attending to the four aspects of dialogic/performative analysis (i.e., temporality, content, context and linguistic narrative positioning) (Riessman 2008). Manual coding of all individualised transcripts was undertaken to initially understand the temporality (i.e., chronology of events), content (i.e., what was said), context (i.e., the environment it related to) work that the narrative was achieving (i.e., the identity that participants were trying to portray through their words) and language used within narratives (i.e., nuances in the language which emphasised or minimised meaning) (Riessman 2008). Attention was given to sense‐making and how participants attributed meaning throughout (Corbally and O'Neill 2014). The second phase focused on identifying narrative patterns common across all six narrative accounts, to determine the existence of common features and codes. Because the focus of the study was to understand life story phases, particular attention was paid to temporality (i.e., noting how the narrative performance of participants' accounts evolved over time) (Riessman 2008).

Throughout both of these phases, a continual collective interpretive analysis process whereby the research team interacted formally, and informally cross‐checking codes, interpretations and findings underpinned both analysis phases. Whilst members of the research team independently participated in data analysis, the underpinning phase of continual collective interpretive analysis (characterised by frequent informal and formal sharing of interpretive findings amongst the group) generated a consensus of findings which form the basis of this paper. Eoin, Daire, Aideen, Claire, Zoe and Aisling were the pseudonyms used for study participants.

## 3 Findings

**FIGURE 1:**
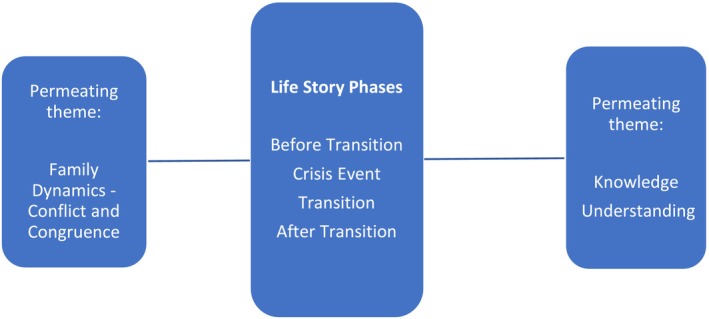
Conceptual representation of study findings.

The participants narrated unique life stories of supporting the transition of their parents into LTC. Although there was some variation in their narrative style and positioning, evidence of commonalities in temporality and identity positioning was evident. In all six cases, four temporal contexts (before transition, crisis event, transition and after transition) were identified as key phases in their life stories. Two themes, family dynamics and knowledge and understanding, influenced all phases of the transitioning process. Figure 1 provides a conceptual representation of the findings.

### 3.1 Life Story Phases—Relatives' Evolving Selves

Each of the four key life story phases demonstrated a clear temporal distinction and an accompanying change in the identity of the participants as they described the varying turning points in their life.

#### 3.1.1 Before Transition

This was the longest transition phase and could last several years. Narrative accounts were dominated by a strong and unshakeable sense of responsibility to care for their parent at home. Some participants positioned this as a historical promise. Eoin had cared for his 94‐year‐old mother for 10 years and reflected:

I did make a … promise that I would never put her into a nursing home unless I felt it was an absolute necessity. (Eoin)

Caring for their parent resulted in sacrifices being made to personal aspects of participants' own lives. In some cases, this work extended into their own retirement years. The gradual progression of caregiving responsibilities may not have been realised although care needs increased over time. This ‘creeping up process’ was evident in all six cases. Daire, Eoin's wife, illustrates how this phenomenon evolved:

Initially, when the caring started 10 years ago it was like quite minor […]. But as the years passed, it just crept up on us so quickly and before we knew it we were 10 years later we didn't realise how bad she was, memory‐wise and physically. […] You still see her mother as she was at 84 whereas now she is 94 and you think she still looks the same for you and mind the same, she still physical capabilities but she's not. (Daire)

Reflecting on her role as sole carer for her mother for over a decade, Aisling recounted her inability to recognise how her mother's care needs had escalated:

I was caring for my mother for five or six years, giving up all my interests and then …maybe for eight or nine years it was a mildly restricted…[when decision was made for 24‐hour care by doctors] it was a huge shock to me because I, I was caring for her and didn't see it in that light. 24‐hour care to me meant drips and changing…. I wasn't expecting that [decision]…I felt I could have kept going…I was kind of just struggling on. (Aisling)

#### 3.1.2 Crisis Event

The key turning point in all life stories was characterised by the ‘crisis event’. For the six participants, this was articulated as sudden and unanticipated. In all cases, it stimulated health professionals' conversations related to LTC admission. The crisis event represented a huge shock and forced relatives to recognise that their promise to care for their loved one was in jeopardy. A loss of control was also perceived by the participants. Zoe highlights the crisis event which led to her mother's admission into hospital and eventual transition into LTC:

It was just fall after fall after fall…Carers ringing me… I was dreading the carer, arriving in the morning to tell me they'd found her fallen…and eventually, on one of the falls, one of the ambulance call outs, the guys actually said, the ambulance crew said ‘this isn't right, this isn't fair. It isn't fair on her, you can't keep going, you can't keep doing this…'. At this stage like, they were turning up so regularly… so, I kind of said, you know, these guys, they are right… We went to see (name of doctor) in the hospital…she was in a wheelchair at that stage we couldn’t, couldn't get her up on her feet, at all. So, we, (Names doctor) said ‘why don't we take her into [LTC], try and get her back on her feet, at least, and then see where we go? (Zoe)

Healthcare professionals were instrumental in suggesting the transition to LTC. Aisling, a single lady in her 60s who was coping with anxiety, reflected on her experience in supporting the transition of her mother (age 84). Her story recalls the multidisciplinary care team meeting which enabled a frank discussion on the reality of her mother's care requirements. This permitted the explicit acknowledgement of Aisling's own experience of coping with care and permitted her to accede to healthcare professionals' advice:

So, at the end of the meeting, they just… said (mother) needs nursing care, 24‐hour….it would be too much for me…I have agreed to nursing home care…the meeting just made life easier for me, you know. I don't think I could have said ‘yes, I want my mother to go into nursing home care’… It was sort of made at the meeting, the decision was made … After the initial meeting, there was the feeling of relief…because I wasn't coping great really as her sole carer. (Aisling)

The involvement of nurses and doctors at this stage was key for the participants, as it ensured that the participants' personal promises (and identity as dutiful carers) with their loved ones remained unbroken as they had supported their parents for as long as possible.

#### 3.1.3 Transition

This life story phase began from the moment the decision was made to transition the older adult into LTC. Sometimes this took weeks, other times, months. Within this phase, participants spoke of their identity transition as they perceived they had relinquished their roles as carers. The participants described this as an emotional time, during which both relief and guilt emerged, either as taking a leap of faith or as being on a mission. For Aideen, who came from a large family that didn't initially agreed on a suitable care location, this mission is reflected in active visiting of potential LTC facilities to assess suitability. However, the placement destination was pre‐empted by the acute care facility, which focused on fitness for medical discharge. In this case, the physical transition was experienced as sudden with the hope that the LTC facility would be appropriate:

Within a week we were told ‘right your mum is ready to go, there is a place. She's going in the next couple of days. It was as sudden as that. We literally had appointments all that week to go and see other, other, nursing homes…. it's a dilemma in say like looking at one nursing home rather, you know, than another. […] It's a bit of a leap of faith no matter where you go… because you don't know. (Aideen)

Although not all participants described this situation the same way, other participants also spoke of getting the LTC facility that was considered best for their parent. Claire described co‐caring with her siblings and was aware of her father's choice of LTC; she described her battle to support his admission into that specific facility. Similar to Aideen, this was positioned as a mission to be undertaken, and she was obliged to lobby for this to be realised.

During the transition, the caring identities of the participants were challenged. As the participants experienced dual emotions of guilt and relief, they struggled to adjust to giving up their community‐based caring role and assuming a new identity. In the narrative below, Aisling navigates her new identity and attempts to maintain continuity in connection through daily visits:

I went in to see her, I started to go in every day, because I think, out of guilt maybe more than, well, love and guilt, but I think they were in equal measure, you know. It's like my role was swept away from me, and I'm looking for it, you know, the role, my role was taken over. (Aisling)

#### 3.1.4 After Transition

Post admission to LTC, the identity transition of participants was most evident in this life story phase. Participants described how they re‐evaluated their lives after transition. Common conflicting emotions expressed during this time included relief, grief and guilt. Aisling ‘could start doing things again’ like going out to concerts, Eoin felt ‘a fantastic relief’ since his mother was safely cared for, allowing him and Daire to travel to their son's wedding, and Claire expressed great relief when her father was admitted and received appropriate care in the nursing home of his choice. Aisling explains the effect of her mother's transition to LTC on her life. Her identity as a carer was replaced with someone who was ‘free’ although that reality was not necessarily experienced:

My life changed totally because, my, my life revolved around her totally, you know. So that even coming home, I remember the first night, it was weird, I still thought she was in the house. […] I have a bit of peace with, you know, the nursing care is great and, I couldn't even, the slightest thing, lift her, move her, I just know I couldn't care for her now at home, myself, you know. So, I think I finally got a kind of a peace about, but it wasn't as easy as I thought because, you think you suddenly you got your freedom and life can suddenly take off but it doesn't… (Aisling)

Yet, the loss of a previous identity and related role was impacted not only by the relief of caregiving responsibilities but by their parent's physical absence in the home leading to loneliness equating to grief:

I kind of think it's like when you go through grief, and you get to the end, and you get to the stage of you actually realise that you're always going to miss the person, you're always going to feel that way that's just the way it's going to be, and that's kind of the acceptance. (Zoe)

Thus, the participants' after‐transition narratives demonstrated a significant impact that reflected feelings of relief, grief and freedom. While the four phases of transition provided a temporal aspect of the findings, two permeating themes (family dynamics and knowledge/understanding) underpinned the collective life stories and sense‐making processes of participants. These are discussed further below.

### 3.2 Family Dynamics—Conflict and Congruence

The theme ‘family dynamics’ was the strongest of the permeating themes. Families were seen as complex, with some demonstrating mutuality in support in both the caregiving role and the transition to LTC. There was evidence of family dynamics having a positive influence on the transition process. Aisling, acknowledged her brother as a ‘head person’ and herself as a ‘heart person’ and was retrospectively grateful for his influence in prompting the transition of their mother to LTC when her own physical and mental health was suffering:

I think (Brother), maybe was more sensible in the sense that he saw reality whereas I just felt bad, and I thought I can't do this…he was quite realistic with me, when he said, ‘I won't be able to help you out that much, you know? I can come up, but I wouldn't be able to be here…’ (Aisling)

However, conflict was also described within families. For example, a lack of support from relatives and siblings was reported by some participants. This created friction and disruption to care continuity, particularly when family members were not previously involved regularly. Eoin, describes his evaluation of his sister's negative intervention in their mother's care:

I've actually called it the seagull school of management, you know. They fly in [sister lives in USA], cause shit all over the place or, you know… And then they fly out again, you know, and to an extent, they'd be upsetting carers…(Eoin)

Similarly, Aideen likened her family dynamic to a ‘war and faction that kept going and going’. This family ‘faction’ was present prior to the transition, hindered the transition process and could continue after transition. Evidence of anticipatory concern for future problems in the LTC facility was also identified given the family's track record of diverging opinions:

It's always been the case, you know… [siblings] who make the phone calls and make complaints and whatever. [siblings] don't come in to see her, but complain otherwise, you know? So, you'd have that fear that, em, you're always under the black cloud of the, the family dynamic. (Aideen)

### 3.3 Knowledge/Understanding

The second permeating theme of ‘knowledge and understanding’ was evident through the life story phases, but the nature of knowledge/understanding seemed to vary over the transition time. Within all narratives, mismatched perceptions regarding the imagined and actual reality of the transitioning process into LTC were evident coupled with their descriptions of a lack of understanding and information. While healthcare providers seemed to play a key role, participants retrospectively acknowledged their own failure to prepare for the eventuality of supporting the transition, which resulted in a stressful and very negative experience for them.

Knowledge deficits were most evident in the pre‐transition phase. A substantial lack of key information rendered the process more traumatic, rushed and confusing. As a result, none of the participants seemed prepared for the transition, despite the fact that the ‘before transition’ time period could span several years. Most participants likened their awareness to a ‘creeping‐up’ process‐a process of slow realisation of the gravity of their situation. This was demonstrated through their use of terms such as ‘I didn't know’ or ‘didn't realise’ to articulate a situation they were somewhat peripherally aware of but unprepared for. Eoin reflects on his story of a changed reality:

I didn't plan it very well and I should have planned things better. I should have realised that em, a day would come when. Mum is age 94, and she was gonna go sooner than rather I should have thought. Em, I didn't really make myself aware […] It was a situation where it was just really reaction to a situation rather than being proactive to a situation. (Eoin)

Accounts of poor communication between healthcare professionals and family members (which emerged as a lack of information/knowledge received) contributed to increased stress and to a negative experience during the ‘crisis event phase’ and ‘transition phase’. Claire articulated this as ‘the not knowing’ period where she felt the multidisciplinary team excluded her from discussions regarding her father's discharge to LTC.

A common preconception perpetuated in advertising was that LTC facilities constituted a home‐from‐home. However, after a traumatic transition, many participants found that such perceptions were mismatched compared to reality. This was influenced by factors such as their parent room‐sharing, a perceived lack of privacy, or their parent being in a plain‐looking room. Aideen reflects on this mismatch of preconception and reality:

We were trying to look for somewhere that was a home from home and I think we were probably naive and we were probably optimistic…things you don't, you don't realise, you know, the doors are open on her room all the time, you know, staff come in and out all the time so…she has progressed dementia but she can be very logical, and she can be very lucid and she can be, she can ask questions and absolutely stump you. And sometimes when she says things like ‘well, do you know who he [nurse] is? He's just after walking in and out of my bedroom’. And he's every right to be there and he's looking after her. It's not a home, it's a very different environment… we did bring in pictures and brought in bits and pieces… that definitely helped. (Aideen)

Other participants voiced confidence in their ‘knowing’ that the transition to LTC was right. This is in stark contrast with the ‘not knowing’ which was associated with the pre‐transition phase and always was expressed in a positive way. This contributed to the happiness of their parent and the participants' confidence in care:

We just knew this was the place for her, there was no smell of nursing home, very efficiently run, the staff were happy, so therefore I knew that (mother‐in‐law) would be happy. (Daire)

So we knew. Yeah, I knew, yeah, yeah, it was a great relief, for all of us that he got in here into (name of ward) from the onset and all his needs and, I mean, it's been fantastic. I mean the care was just fantastic cause every, I mean the care all along was fantastic. (Claire)

Thus, participants reflected on a complex navigation of understanding and knowledge, where there was a temporal element in their experiences. Before the transition to LTC, they could express a lack of knowledge regarding the ‘true’ life world of communal living. However, this may be linked to the other findings where the transition was experienced as unanticipated and complicated by feelings of grief and letting the older person down. Paradoxically, as time progressed and the older person settled in a desirable LTC facility, participants could express relief and contentment that this was a positive move.

## 4 Discussion

The risk of multi‐morbidity increases as people age (Salive 2013). While many health systems promote primary healthcare and ageing in place (CDC 2009), when caregiving demands increase, a transition to a LTC facility is a decision considered in the context of needs, safety and quality of life. While this choice is one that the older person with decision‐making capacity makes, there are significant impacts on informal carers. Supporting a decision to transition a loved one is fraught with emotions and is an important aspect of gerontological and community nursing (Egan et al. 2022; O'Neill et al. 2020; Zamanzadeh et al. 2017). A consciousness by the nurse of the particular life phase identified by this study is argued to be crucial in providing appropriate support and education in response. Globally, statistics on carers are similar to that of nurses with most carers being female family members (Petrillo, Bennett, and Pryce 2022; Carers Australia 2022; Family Carers Ireland 2022; Carers' UK 2022). This disproportionate genderised role in caregiving is reflected in the participants in this study and perhaps reflect societal expectations of female relatives within family structures.

This narrative study demonstrates that participants' stories are immersed in emotional ties with the older person and may be contextualised in wider supportive families or family conflict. This study highlights how four key life story phases related to the significant life event of transitioning an older person to LTC. Holstein and Gubrium (2000) highlight how societal structures and personal agency inform the portrayal and ‘transitions of self’ in naturally evolving narratives to that which best portray a socially desirable identity. While previous reassurances and promises may have been made to care for the parent at home, this commitment was superseded by increasing care demands. This finding reflects the moral responsibility within family expectations of reciprocity (Liken 2001; Bradley Bursack 2009) also reflected in the Confucian concept of filial piety (Ramanathan and Fisher 2016) as well as awareness of dominant desired public identities (i.e., an attentive caregiver) in informing how participants account for themselves through their life story (Holstein and Gubrium 2000). Community nurses with a gerontological focus in particular have the potential to make a real difference in supporting relatives who may struggle with such challenges.

Similar to findings from Magilvy and Congdon (2000) and Merla et al. (2018), participants' transitioning process was precipitated by a crisis event that disrupted their caring ability. Even though a parent's decline may have been apparent, its gradual nature could mask recognition. Consequently, participants were unprepared when the crisis event happened and perceived a loss of control of the situation. Simultaneous knowing and not knowing about the realities of a grave situation has been identified as a tactic used to buffer reality (Cohen 2010). When the older person was admitted to acute care, the participants reported feeling their caring role and decision‐making tacitly transferred to the healthcare professionals. Ryan and Scullion (2000) support this finding, also recognising that relatives can resist LTC admission until there is no other option. However, the instrumentality of healthcare professionals in initiating discussion and facilitating shared decision‐making processes between caregivers, older persons and professionals in relation to transitioning to LTC highlights the crucial role gerontological nurses and nurses working in the community with a gerontological focus in particular play in supporting families at this challenging time in the life course when family members may not have fully appreciated the realities of the situation. Shared decision‐making between older people, family and nursing home staff is linked to minimising communication errors, can increase the autonomy of residents (Cranley et al. 2020) and is aligned with a person‐centred, human rights approach (Phelan 2020). Like the narratives in this study, where only one parent's will and preference were spoken of, Rhynas et al. (2018) observe the general absence of the older person's voice in transition decisions. However, it was noted that several participants' parents were living with dementia. Yet, outside of decision‐making capacity challenges, contributing factors to a lack of voice include complex cultural misperceptions of next of kin's authority in decision‐making, professional power subservience and ageism (Polacsek and Woolford 2022). It is crucial that gerontological and community nurses remain cognisant of these issues as relatives navigate their own positions throughout each life story phase.

After the transition occurred, participants had to re‐evaluate their own positions and identities. As Oyserman (2001) observes, identity is dynamic, mediated by social production through the cognitive self, context and position. This was particularly evident after transition where carers no longer positioned identified themselves as caregivers because of the fact that caregiving activities were assumed by staff in the LTC facility. However, meaningful involvement by gerontological nursing staff as well as other healthcare providers to enable families to continue in a caregiving role can increase the quality of life for the older resident (Restorick Roberts and Ishler 2018; Puurveen, Baumbusch, and Gandhi 2018). This suggests that supporting a revision of roles towards a collaboration between informal and formal caregivers can support a continued carer identity. Stories included accounts of abandoning previous carer roles and sense‐making in readjusting to their new reality. The transitioning of self was also evidenced through opposing narratives describing pendulating emotions of guilt and relief. While ‘carer’ relief from primary activities due to LTC admission has been noted in other studies, stress levels can persist (Zarit and Whitlatch 1993) and as demonstrated by the participants, could be aggravated by unhelpful healthcare professionals. However, as reflected in the findings and elsewhere (Martz and Morse 2016; Graneheim, Johansson, and Lindgren 2013), visiting their older relatives aided their life readjustment transition. While feeling guilt related to LTC admission is not uncommon (Prunty and Foli 2019), in contrast to Gallego‐Alberto et al. (2022), some participants in this study demonstrated a reduction of guilt feelings over time. To some extent, this was mediated by a desire to select an LTC facility that was deemed suitable and where they perceived their older relative would be happy. Despite this desire, participants observed that their home‐from‐home perceptions of life in LTC could be misplaced. Previous research has shown that relatives' perception of the characteristics of the facility influenced their views on the quality of life in LTC (Ryan and McKenna 2015; Restorick Roberts and Ishler 2018).

Family dynamics provided the conditions of experience and identity in how the transition was experienced by participants. This theme describes how inter‐familiar relations strongly contributed to the outcomes of the decision‐making process. Family members spoke of altruism as they strove to support their older parents, particularly in the context of an older person living with dementia. While some participants described support from siblings and other family members in navigating the transition, others experienced conflict evidenced, for example, by descriptions of different intra‐family opinions on what was best for their older relative. Conflicts and discordance between family members in the choice of LTC are not uncommon (Cheek and Ballantyne 2001) and this could disrupt the decision‐making process and delay progress in the transition. Awareness by gerontological nurses of this potential cause of progress delay has the potential to assist their interventions in diffusing potential conflicts in supporting the transition process. Family conflict is also associated with a primary carer experiencing stress secondary to role strain (Polenick and DePasquale 2019). The stories presented by some participants support this, as they attempted to navigate care, in the context of divergent and sometimes acrimonious siblings' input.

The theme of ‘knowledge and understanding’ determined the outcome of their whole transitioning experience. This may be intricately linked to not anticipating the transition. All participants lacked knowledge and information about LTC, and how to smoothly support the transition of an older relative, especially when dementia was a factor. This study highlights missed opportunities within the community setting where nurses and healthcare providers could potentially reduce knowledge deficits, creating a space for caregivers and older persons to facilitate discussions regarding possible mechanisms to enhance the quality of life within the home (perhaps introducing daycare options or suggesting additional appropriate supports) rather than waiting for a crisis to occur. Attention by gerontological nurses to the importance of sequencing information appropriate to the particular life phase would be useful for caregivers to assist in meeting particularised information deficits. Initiation and utilisation of caregiver stress inventories might prove useful as a screening instrument but also to aid the prompting such discussions in the context of community care. Reduction of knowledge deficits and enabling families to live well whilst being prepared and informed prior to a crisis event is important.

Family members spoke of a lack of information and guidance in selecting appropriate LTC facilities which met specific care needs. Accordingly, not knowing contributes to a lack of preparedness, hindering the process and engendering a more traumatic experience (Johansson et al. 2014). Consequently, family members can be unprepared for their relatives' transition to LTC (Nolan and Dellasega 2000; Eika et al. 2014). Furthermore, having little knowledge and preparation, some of the participants found their preconceptions of the nursing home environment (i.e., privacy and homeliness) were not as they had perceived from the little information they had gathered prior to the transfer. These findings illuminate the lack of support for carers in navigating necessary key information for transitions leading to stories reflecting this as a traumatic experience. Consequently, even though there were stories of losing control of the situation to healthcare professionals, having more support and being able to discuss the environment of LTC with them would alleviate the related stress. Other strategies, such as supporting standardised user‐friendly documentation can contribute to person‐centred transition experiences for all stakeholders (O'Reilly et al. 2019).

### 4.1 Strength and Limitations

This qualitative study examined narrated accounts of six family members' life stories of supporting the transition of a parent to LTC. While generalisability was not a focus, even with a small sample size, there is potential for transferability of findings. The narratives produced in‐depth stories of the carers' experiences of LTC transitions, underpinned by a case‐oriented, information‐rich analysis (Sandelowski 1996). Whilst narrative approaches enable a ‘storied way of knowing and communicating’ (Riessman 2005: 1), the dialogic/performative analytic framework used in this study allowed for a multidimensional approach to narrative analysis which explored thematic, structural, interactional and performative aspects as well as elucidating a temporal journey of a significant life change and identity transformation. Using the Biographical Narrative Method of interviewing enabled the development of the participants' lived experience via ‘whole stories’ (Wengraf 2008: 4) who told their accounts without interruption. A limitation of the study is that participants were also all second‐generation relatives, thus the variability of experience of other carers' (spouse and sibling) navigation of LTC transitions may differ. Moreover, a wider representation of recruitment sites could identify formal support differences which may impact carers' stories.

## 5 Conclusion

Attending to stories and how individuals make sense of their experiences is an aspect of gerontological nursing assessment and it could be argued that the quality of nursing care is influenced by the quality of interpretation of such stories. Narrative approaches resonate with the practice of nursing (Corbally and O'Neill 2014) and provide ideal conditions to research how individuals make sense of their experiences over time through situated, contextualised identities. Understanding small stories and how individuals position themselves over time also indirectly assists those in healthcare to understand how societal norms and expectations impact on what individuals feel safe to disclose about themselves in their society (Potter and Wetherell 1987). The findings presented in this study are ‘worthy’ small stories insofar as they belong on the fringes of the mainstream practice which typically focuses on the ‘older relative’ and not necessarily on the life story of the relatives (Bamberg and Georgakopoulou 2008). Concurrently, the study emphasises the gap in health care in the context of person‐centred health care that incorporates family members' experiences at critical points of care transition processes. The implications of this study for research highlight the resonance of narrative methods in generating new and nuanced knowledge regarding how life stories evolve over time.

The four life story phases identified (before transition, crisis event, transition and after transition) provide a helpful means for gerontological nurses to recognise the sequential challenges faced by family members and caregivers as well as highlighting the varying time duration of each phase and varying intensity of potential nursing involvement depending on each phase. While some research has explored the experiences of carers in transitions to LTC, knowledge regarding family members remains relatively scant. This study helps contribute to knowledge in this area and highlights the need for a temporal approach to carers as client reinforcing the need to align with international policy initiatives which foster and support ongoing informal care mechanisms to promote living well in their own environments for as long as sustainably possible (Leichsenring et al. 2013; Centre for Policy on Ageing 2014). Within the diversity of nursing settings, the findings illustrate the potential for gerontological nurses to deliver anticipatory guidance and support on related care implications for older persons and their relatives. In acute care, there is potential to provide support and information on pragmatic pathways of care and giving information on specific care needs may assist in identifying suitable placements. In LTC facilities, gerontological nurses can negotiate meaningful involvement of family members in care. Knowledge and understanding can also be augmented by sequencing of information related to the particularised life story phase of the older person to alleviate potential knowledge deficits at all phases. These can be complimented by plain language information guides for additional reference and support. The crucial role of gerontological nurses in creating a climate of facilitative care amongst older people and their relatives cannot be overstated. We are hopeful that the study findings illustrate how and when nurses can best potentiate the provision of information, support and guidance to all within their care.

## Author Contributions

Study design: M.C., D.R., O.F.; Data Collection: O.F., R.R.; Data Analysis: M.C., R.R., O.F., D.R., A.P.; Manuscript Preparation; M.C., D.R., O.F., R.R., A.P.

## Ethics Statement

Institutional ethical approval for the study was granted by the Research Ethics Committee at Dublin City University in March 2017 (Reference: DCUREC/2016/207).

## Conflicts of Interest

The authors declare no conflicts of interest.

## Data Availability

The data that support the findings of this study are available on request from the corresponding author. The data are not publicly available due to privacy or ethical restrictions.

## References

[opn70001-bib-0001] Abdi, S. , A. Spann , J. Borilovic , L. de Witte , and M. Hawley . 2019. “Understanding the Care and Support Needs of Older People: A Scoping Review and Categorisation Using the WHO International Classification of Functioning, Disability and Health Framework (ICF).” BMC Geriatrics 19: 195. 31331279 10.1186/s12877-019-1189-9 PMC6647108

[opn70001-bib-0002] Argyle, E. , M. Downs , and J. Tasker . 2010. “Continuing to Care for People With Dementia: Irish Family Carers' Experience of Their Relative's Transition to a Nursing Home.” Research Report. https://hdl.handle.net/10147/141260.

[opn70001-bib-0003] Bamberg, M. , and A. Georgakopoulou . 2008. “Small Stories as a New Perspective in Narrative and Identity Analysis.” Text & Talk ‐ An Interdisciplinary Journal of Language Discourse Communication Studies 28, no. 3: 377–396.

[opn70001-bib-0004] Berger, P. L. , and T. Luckmann . 1966. The Social Construction of Reality. London: Penguin Books.

[opn70001-bib-0005] Bradley Bursack, C. 2009. “I Promised My Parents I'd Never Put Them in a Nursing Home.” https://www.agingcare.com/Articles/I‐promised‐my‐parents‐I‐d‐never‐put‐them‐in‐a‐nursing‐home‐133904.htm.

[opn70001-bib-0006] Carers' Australia . 2022. “Who Is a Carer?” https://www.carersaustralia.com.au/about‐carers/who‐is‐a‐carer/.

[opn70001-bib-0007] Carers UK . 2022. Key Facts. London: Carers UK.

[opn70001-bib-0060] Casey, B. , D. Proudfoot , and M. Corbally . 2016. “Narrative in Nursing Research: An Overview of Three Approaches.” Journal of Advanced Nursing 72, no. 5: 1203–1215. 26749518 10.1111/jan.12887

[opn70001-bib-0008] Centers for Disease Control and Prevention (CDC) . 2009. “Healthy Places Terminology.” https://www.cdc.gov/healthyplaces/terminology.htm.

[opn70001-bib-0009] Centre for Policy on Ageing . 2014. “Rapid Review: The Care and Support of Older People: An International Perspective.” http://www.cpa.org.uk/information/reviews/CPA‐Rapid‐Review‐The‐care‐and‐support‐of‐older‐people‐an‐international‐perspective.pdf.

[opn70001-bib-0010] Cheek, J. , and A. Ballantyne . 2001. “Moving Them on and in: The Process of Searching for and Selecting an Aged Care Facility.” Qualitative Health Research 11, no. 2: 221–237. 11221117 10.1177/104973201129119064

[opn70001-bib-0011] Cohen, S. 2010. States of Denial: Knowing About Atrocities and Suffering. Cambridge: Polity Press.

[opn70001-bib-0012] Corbally, M. A. , and C. O'Neill . 2014. “An Introduction to the Biographical Narrative Interpretive Method.” Nurse Researcher 21, no. 5: 34–39. 10.7748/nr.21.5.34.e1237 24877909

[opn70001-bib-0013] Cranley, L. A. , S. E. Slaughter , S. Caspar , et al. 2020. “Strategies to Facilitate Shared Decision‐Making in Long‐Term Care.” International Journal of Older People Nursing 15, no. 3: e123. 10.1111/opn.12314 PMC7507187 32196984

[opn70001-bib-0014] Egan, C. , C. Naughton , M. Caples , and H. Mulcahy . 2022. “Shared Decision‐Making With Adults Transitioning to Long‐Term Care: A Scoping Review.” International Journal of Older People Nursing 18, no. 1: e12518. 36480119 10.1111/opn.12518 PMC10078233

[opn70001-bib-0015] Eika, M. , G. A. Espnes , O. Söderhamn , and S. Hvalvik . 2014. “Experiences Faced by Next of Kin During Their Older Family Members' Transition Into Long‐Term Care in a Norwegian Nursing Home.” Journal of Clinical Nursing 23, no. 15–16: 2186–2195. 24372931 10.1111/jocn.12491

[opn70001-bib-0016] Family Carers Ireland . 2022. Caring Through Covid. Dublin: FCI.

[opn70001-bib-0017] Gallego‐Alberto, L. , H. J. Smaling , A. L. Francke , T. van de Brug , J. T. van der Steen , and K. J. Joling . 2022. “The Relationship Between Guilt Feelings, Conflicts With Staff and Satisfaction With Care in Relatives of Nursing Home Residents With Dementia: A Longitudinal Analysis.” Dementia 1: 5–20. 10.1177/14713012211024015 34250841

[opn70001-bib-0061] Goffman, E. 1959. The Presentation of Self in Everyday Life. New York: Doubleday.

[opn70001-bib-0018] Graneheim, U. H. , A. Johansson , and B. M. Lindgren . 2013. “Family Caregivers' Experiences of Relinquishing the Care of a Person With Dementia to a Nursing Home: Insights From a Meta‐Ethnographic Study.” Scandinavian Journal of Caring Sciences 28, no. 2: 215–224. 23578033 10.1111/scs.12046

[opn70001-bib-0019] Holstein, J. A. , and J. F. Gubrium . 2000. The Self We Live by – Narrative Identity in a Postmodern World. Oxford: Oxford University Press.

[opn70001-bib-0020] Johansson, A. , H. O. Ruzin , U. H. Graneheim , and B. M. Lindgren . 2014. “Remaining Connected Despite Separation – Former Family Caregivers' Experiences of Aspects That Facilitate and Hinder the Process of Relinquishing the Care of a Person With Dementia to a Nursing Home.” Aging & Mental Health 18, no. 8: 1029–1036. 24807210 10.1080/13607863.2014.908456

[opn70001-bib-0021] Kalache, A. , S. M. Barreto , and I. Keller . 2005. “Global Ageing: The Demographic Revolution in all Cultures and Societies.” In The Cambridge Handbook of Age and Ageing, edited by M. Johnson , 30–46. Cambridge: Cambridge University Press.

[opn70001-bib-0022] Kane, R. L. , and R. A. Kane . 2005. “Long Term Care.” In The Cambridge Handbook of Age and Ageing, edited by M. Johnson , 638–646. Cambridge: Cambridge University Press.

[opn70001-bib-0023] Kong, Y. L. , J. Anis‐Syakira , S. Jawahir , Y. R'ong Tan , N. H. A. Rahman , and E. H. Tan . 2021. “Factors Associated With Informal Caregiving and Its Effects on Health, Work, and Social Activities of Adult Informal Caregivers in Malaysia: Findings From the National Health and Morbidity Survey 2019.” BMC Public Health 21: 1033. 34074275 10.1186/s12889-021-11022-1 PMC8170800

[opn70001-bib-0062] Leichsenring, K. , H. Nies , and R. van der Veen . 2013. “The Quest for Quality in Long‐Term Care.” In Long‐Term Care in Europe, edited by K. Leichsenring , J. Billings , and H. Nies . London: Palgrave Macmillan.

[opn70001-bib-0024] Liken, M. A. 2001. “Managing Transitions and Placement of Caring for a Relative With Alzheimer's Disease.” Home Health Care Management & Practice 14, no. 1: 31–39.

[opn70001-bib-0025] Lowenstein, A. 2005. “Global Ageing and Challenges to Families.” In The Cambridge Handbook of Age and Ageing, edited by M. Johnson , 403–412. Cambridge: Cambridge University Press.

[opn70001-bib-0026] Magilvy, J. K. , and J. G. Congdon . 2000. “The Crisis Nature of Health Care Transitions for Rural Older Adults.” Public Health Nursing 17, no. 5: 336–345. 11012996 10.1046/j.1525-1446.2000.00336.x

[opn70001-bib-0027] Martz, K. , and J. M. Morse . 2016. “The Changing Nature of Guilt in Family Caregivers: Living Through Care Transitions of Parents at the End of Life.” Qualitative Health Research 27, no. 7: 1006–1022. 27206457 10.1177/1049732316649352

[opn70001-bib-0028] Merla, C. , A. Wickson‐Griffiths , S. Kaasalainen , et al. 2018. “Perspective of Family Members of Transitions to Alternative Levels of Care in Anglo‐Saxon Countries.” Current Gerontology and Geriatrics Research 2018: 1–11. 10.1155/2018/4892438 PMC5841098 29681932

[opn70001-bib-0029] Nolan, M. , and C. Dellasega . 2000. ““I Really Feel I've Let Him Down”: Supporting Family Carers During Long‐Term Care Placement for Elders.” Journal of Advanced Nursing 31, no. 4: 759–767. 10759971 10.1046/j.1365-2648.2000.01346.x

[opn70001-bib-0030] O'Neill, M. , A. Ryan , A. Tracey , and L. Laird . 2020. ““You're at Their Mercy”: Older Peoples' Experiences of Moving From Home to a Care Home: A Grounded Theory Study.” International Journal of Older People Nursing 15: e12305. 31997550 10.1111/opn.12305

[opn70001-bib-0031] O'Reilly, P. , B. O'Brien , M. M. Graham , et al. 2019. “Key Stakeholders' Perspectives on the Development of a National Transfer Document, for Older Persons, When Transferring Between the Residential and Acute Care Settings: A Qualitative Descriptive Study.” International Journal of Older People Nursing 14, no. 4: e12254. 31347762 10.1111/opn.12254

[opn70001-bib-0032] Orem, D. 2001. Nursing: Concepts of Practice. New York: Mosby.

[opn70001-bib-0033] Oyserman, D. 2001. “Self‐Concept and Identity.” In The Blackwell Handbook of Social Psychology, edited by A. Tesser and N. Schwarz , 499–517. Malden, MA: Blackwell.

[opn70001-bib-0034] Petrillo, M. , M. Bennett , and G. Pryce . 2022. “Cycles of Caring: Transitions in and Out of Unpaid Care.” https://centreforcare.ac.uk/updates/2022/11/new‐report‐carers‐rights‐day‐2022/.

[opn70001-bib-0035] Phelan, A. 2020. Advances in Elder Abuse Research: Practice, Legislation and Policy. First, United Kingdom: Springer.

[opn70001-bib-0036] Polacsek, M. , and M. Woolford . 2022. “Strategies to Support Older Adults' Mental Health During the Transition Into Residential Aged Care: A Qualitative Study of Multiple Stakeholder Perspectives.” BMC Geriatrics 22: 151. 35209848 10.1186/s12877-022-02859-1 PMC8866554

[opn70001-bib-0037] Polenick, C. A. , and N. DePasquale . 2019. “Predictors of Secondary Role Strains Among Spousal Caregivers of Older Adults With Functional Disability.” Gerontologist 59, no. 3: 486–498. 29325105 10.1093/geront/gnx204 PMC6784276

[opn70001-bib-0038] Potter, J. , and M. Wetherell . 1987. Discourse and Social Psychology. Beyond Attitudes and Behavior. London, UK: Sage.

[opn70001-bib-0039] Prunty, M. M. , and K. J. Foli . 2019. “Guilt Experienced by Caregivers to Individuals With Dementia: A Concept Analysis.” International Journal of Older People Nursing 14, no. 2: e12227. 30793838 10.1111/opn.12227

[opn70001-bib-0040] Puurveen, G. , J. Baumbusch , and P. Gandhi . 2018. “From Family Involvement to Family Inclusion in Nursing Home Settings: A Critical Interpretive Synthesis.” Journal of Family Nursing 24, no. 1: 60–85. 29455580 10.1177/1074840718754314 PMC5833026

[opn70001-bib-0041] Quinn, C. , and G. Toms . 2018. “Influence of Positive Aspects of Dementia Caregiving on Caregivers' Well‐Being: A Systematic Review.” Gerontologist 59, no. 5: e584–e596. 10.1093/geront/gny168 30597058

[opn70001-bib-0042] Ramanathan, R. , and P. Fisher . 2016. “Singaporean Caregivers' Experiences of Placing a Relative Into Long Term Care.” Clinical Gerontologist 39, no. 3: 167–189.

[opn70001-bib-0043] Restorick Roberts, A. , and K. J. Ishler . 2018. “Family Involvement in the Nursing Homes and Perceived Resident Quality of Life.” Gerontologist 58, no. 6: 1033–1043. 28977636 10.1093/geront/gnx108

[opn70001-bib-0044] Rhynas, S. J. , A. Garcia Garrido , J. K. Burton , G. Logan , and J. MacArthur . 2018. “New Care Home Admission Following Hospitalisation: How Do Older People, Families and Professional Make Decisions About Discharge Information? A Case Study Analysis.” International Journal of Older People Nursing 13, no. 3: e12192. 29573561 10.1111/opn.12192

[opn70001-bib-0045] Riessman, C. K. 2005. “Narrative Analysis.” In Narrative, Memory & Everyday Life, 1–7. Huddersfield: University of Huddersfield.

[opn70001-bib-0046] Riessman, C. K. 2008. Narrative Methods for the Human Sciences. Thousand Oaks, CA: Sage Publications, Inc.

[opn70001-bib-0047] Ryan, A. , and H. McKenna . 2015. ““It's the Little Things That Count”. Families Experience of Roles, Relationships and Quality of Care in Nursing Homes.” International Journal of Older People Nursing 10: 38–47. 24814052 10.1111/opn.12052

[opn70001-bib-0048] Ryan, A. A. , and H. F. Scullion . 2000. “Nursing Home Placement: An Exploration of the Experiences of Family Carers.” Journal of Advanced Nursing 32, no. 5: 1187–1195. 11115004 10.1046/j.1365-2648.2000.01589.x

[opn70001-bib-0049] Sacco, L. B. , S. König , H. Westerlund , and L. G. Platts . 2022. “Informal Caregiving and Quality of Life Among Older Adults: Prospective Analyses From the Swedish Longitudinal Occupational Survey of Health (SLOSH).” Social Indicators Research 160: 845–866.

[opn70001-bib-0050] Salive, M. E. 2013. “Multimorbidity in Older Adults.” Epidemiological Review 35: 75–83. 10.1093/epirev/mxs009 23372025

[opn70001-bib-0051] Sandelowski, M. 1996. “One Is the Liveliest Number: The Case Orientation of Qualitative Research.” Research in Nursing & Health 19, no. 6: 525–529. 8948406 10.1002/(SICI)1098-240X(199612)19:6<525::AID-NUR8>3.0.CO;2-Q

[opn70001-bib-0052] Victor, S. 2009. “Telling Tales: A Review of C. K. Riessman Narrative Methods for Methods for the Human Sciences.” Qualitative Report 14, no. 3: 172–176.

[opn70001-bib-0053] Wengraf, T. 2001. Qualitative Research Interviewing – Biographic Narratives and Semi‐Structured Methods. London: Sage Publications.

[opn70001-bib-0054] Wengraf, T. 2008. “Biographic‐Narrative Interpretive Method (BNIM) for Researching Lived Experience and Whole Lives: A Summary.” https://uel.ac.uk/sites/default/files/interviewing‐for‐life‐histories‐lived‐situations‐and‐personal‐experience‐the‐biographic‐narrative‐interpretive‐method‐bnim‐on‐its‐own‐and‐as‐part‐of‐a‐multi‐method‐full‐spectrum‐psycho‐societal‐methodology.pdf.

[opn70001-bib-0055] Wengraf, T. 2014. “Interviewing for Life‐Histories, Lived Periods and Situations, and Ongoing Personal Experiencing Using the Biographic Narrative Interpretive Method (BNIM): The BNIM Short Guide Bound With the BNIM Detailed Manual.” https://is.muni.cz/el/1423/podzim2014/SOC932/um/Wengraf_manual.pdf.

[opn70001-bib-0056] World Health Organisation . 2022. “Ageing and Health.” https://www.who.int/news‐room/fact‐sheets/detail/ageing‐and‐health#:~:text=At%20this%20time%20the%20share,2050%20to%20reach%20426%20million.

[opn70001-bib-0057] Yuan, Q. , Y. Zhang , E. Samari , et al. 2023. “Positive Aspects of Caregiving Among Informal Caregivers of Persons With Dementia in the Asian Context: A Qualitative Study.” BMC Geriatrics 23: 51. 36707781 10.1186/s12877-023-03767-8 PMC9883086

[opn70001-bib-0058] Zamanzadeh, V. , A. Rahmani , V. Pakpour , L. L. Chenoweth , and E. Mohammadi . 2017. “Psychosocial Changes Following Transition to an Aged Care Home: Qualitative Findings From Iran.” International Journal of Older People Nursing 12: e12130. 10.1111/opn.12130 27709808

[opn70001-bib-0059] Zarit, S. H. , and C. J. Whitlatch . 1993. “The Effects of Placement in Nursing Homes on Family Caregivers: Short and Long Term Consequences.” Irish Journal of Psychology 14, no. 1: 25–37.

